# User Centered Design of Interaction Techniques for VR-Based Automotive Design Reviews

**DOI:** 10.3389/frobt.2019.00013

**Published:** 2019-03-21

**Authors:** Matthias de Clerk, Manfred Dangelmaier, Gernot Schmierer, Dieter Spath

**Affiliations:** ^1^BMW Group, Munich, Germany; ^2^Fraunhofer Institut für Arbeitswirtschaft und Organisation, Stuttgart, Germany

**Keywords:** user centered design, user study, interaction techniques, virtual reality, automotive design

## Abstract

The exterior design is one of the most important selling propositions in the automotive premium market. Because of progressing digitization in the automotive industry, it is increasingly assessed using virtual 3D models. In this context, Virtual Reality (VR) is a key technology of continuously growing importance. However, complicated interaction in VR proves to be a major drawback in industrial settings. In this paper, we report insights of our user centered approach aiming at appropriate VR interaction techniques supporting designers, engineers, and management executives optimally in design assessment. Our approach splits into two iterations according to the main interaction tasks Visual Inspection and Model Comparison. In each iteration, alternative interaction techniques were conceptualized, implemented as prototypes, and evaluated in a user study in terms of Usability, User Experience, Intuitiveness and Task Load. In the first iteration six interaction techniques for Visual Inspection, two speech-based, two gesture-based, and two touch-based variants, were studied. Incorporating the results, the second iteration explores three interaction techniques for Model Comparison utilizing (1) a portable touch remote, (2) hand and body gestures, and (3) a multimodal mix of both. The final concepts yielded high ratings by the participants, but showed significant differences between rational and emotional aspects. We conclude that the acceptance of VR in automotive design could be facilitated by refining and applying these interaction techniques.

## Introduction

Digitization has made good progress in product development in the automotive industry in the last decades. This holds in particular true for workflows in designing vehicle components based on primitive geometries. In contrast, exterior and interior stylists work with complicated free-form surfaces. Therefore, they prefer a more physical approach using tapes to create shapes in 2D and clay material to form 3D models. In recent years there is a strong trend to leave the world of physical modeling as early as possible in the styling process and to create digital models even from the scratch. In consequence, industrial designers and deciders are losing the physical experience of the models. Virtual Reality (VR) shall bring such a close-to-live vehicle experience back again. This paper deals with the question, how vehicle stylists and deciders want and shall interact with such digital models in future in a natural and acceptable way. We consider usable interaction techniques as key success factors for VR in automotive design (Spath and Weisbecker, 2013; Gabbard, 2015).

Our approach is structured in two iterations. First, we conceptualize, implement and evaluate interaction techniques to enable users to visually inspect 3D vehicle exteriors. Second, we enhance these concepts to additionally allow for comparing different exterior variants.

In the following, we provide background of VR-based Automotive Design Reviews, Interaction Techniques and User Centered Design.

### VR-Based Automotive Design Review

**Figure 1:**
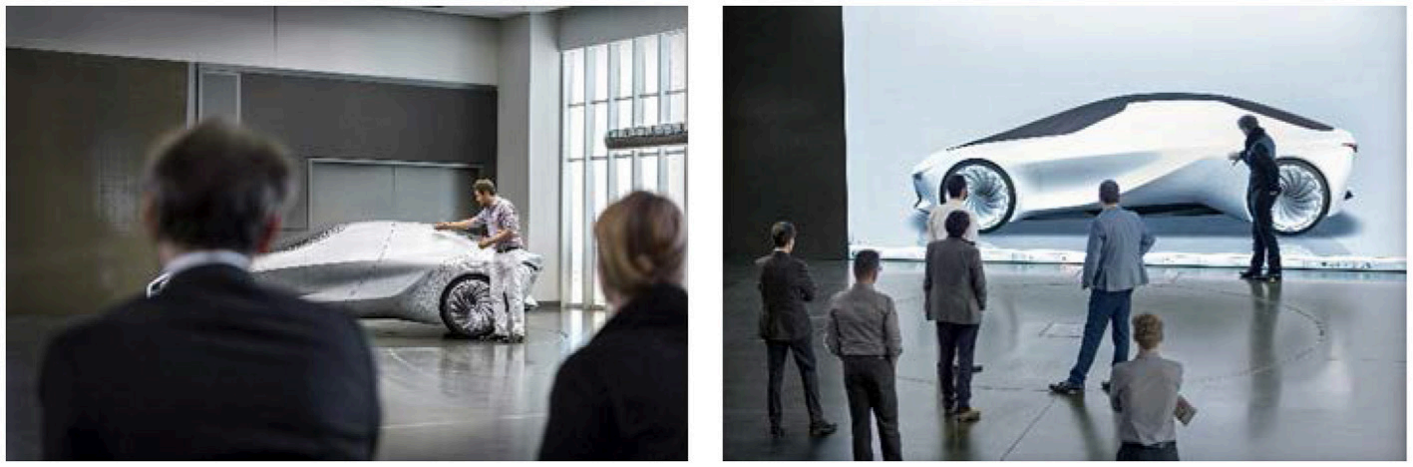
Design Review of the BMW Vision 100 show car using a physical 1:1 model **(Left)** and a VR-based model **(Right)**. © Copyright BMW AG, München.

Automotive design still depends on physical scale 1:1 models that vary from simple mock-ups to fully functional prototypes (Perini, 2007). They enable a profound product experience for important design decisions, but consume many resources. In contrast, virtual models allow for shorter design cycles, less cost, and improved integration of process partners. Hence, automotive design shifts toward the virtual domain (Aust et al., 2011; Maier and Beier, 2011). To foster a reliable product experience using digital data, manufacturers make use of the newest high-end VR technology (Küderli, 2007). Particularly systems based on large high resolution displays (e.g., powerwall) are widely used, since they facilitate photorealistic 1:1 visualizations and collaborative work (Zimmermann, 2008). But operating these systems requires prior knowledge, so design professionals must be supported by VR specialists (Rademacher, 2014). Figure 1 shows a physical and a virtual Design Review using the example of the BMW Vision 100 project.

### Interaction Techniques

Interaction techniques in VR are methods to accomplish tasks (LaViola et al., 2017). They are often categorized according to so-called “universal interaction tasks,” which are navigation, selection, manipulation, system control, and symbolic input (McMahan et al., 2015). From a technical perspective, interaction techniques are also categorized according to the interaction modality, which can be defined as a sensory communication channel between a human and a computer. Common modalities for humans to input information into VR systems are haptic devices (e.g., gamepads, flysticks, touchscreens, special purpose devices), speech input, and gesture recognition (Jerald, 2016). However, HCI research does not provide standards or guidelines for designing interaction techniques for such a vehicle concept experience scenario (Dangelmaier, 2009). As a result, we focus on creating interaction techniques that enable adequate interactions with 3D vehicle exteriors based on those input modalities for large screen VR systems.

### User Centered Design

User Centered Design (UCD) is a systematic method resulting in usable products, systems or services by explicitly focusing on the users (ISO 9241–10, 2010). Traditionally, the primary goal is the maximization of Usability, which is formally defined as the extent to which a product can be used by specified users to achieve specified goals with effectiveness, efficiency and satisfaction in a specified context of use (ISO 9241–10, 2010). Today, recent research adopts a broader perspective, the User Experience, which includes all human perceptions and responses resulting from the use and anticipated use (ISO 9241–210, 2010). For our solution approach we additionally consider the aspects Intuitiveness (Blackler et al., 2006; Blackler and Hurtienne, 2007; Naumann et al., 2007) and Task Load (Szalma et al., 2012) to obtain a comprehensive view of the perceived usage quality.

The UCD process is iterative and consists of four phases (ISO 9241–210, 2010). The first phase begins with the analysis of the context of use including users, goals, tasks and environments. In the second phase, the requirements of use are derived from the analysis. The third phase continues with the development of alternative design solutions. In the fourth phase, an evaluation is carried out and the procedure is repeated until the design solutions satisfy the requirements of use. The process is generic and can be adapted to the development of VR experiences (Jerald, 2016). In this paper, we emphasize the development (phase three) and evaluation (phase four) of interaction techniques within our iterative approach.

## Iteration 1: Interaction Techniques for Visual Inspection

In the first iteration, we developed interaction techniques for Visual Inspection and implemented a high fidelity prototype (de Clerk et al., 2017). Using the prototype, we conducted a user study in order to gain empirical insight into how potential users experience the usage quality of the different interaction concepts.

### Concept and Implementation

#### Concept

**Figure 2:**
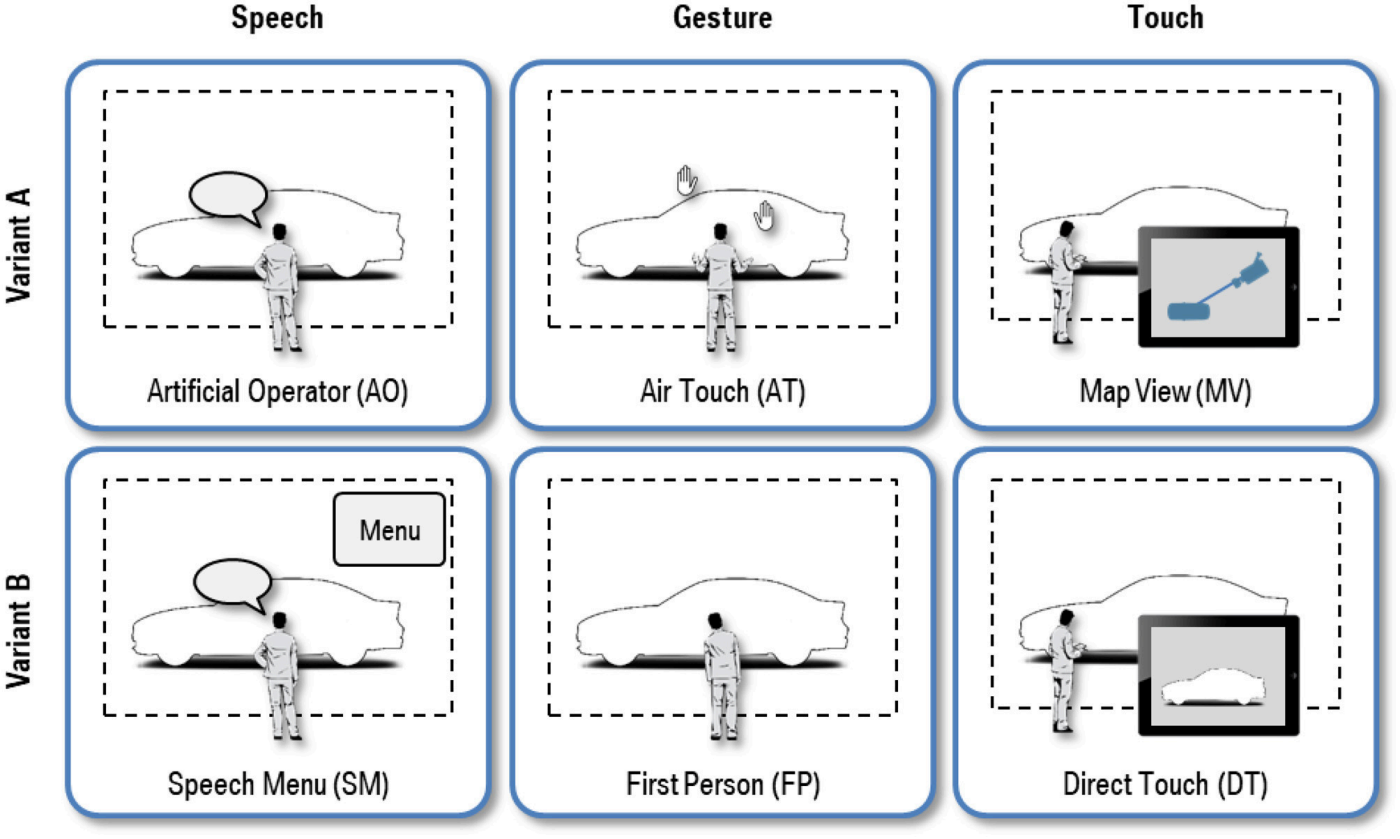
Interaction techniques for Visual Inspection of 3D vehicle exteriors.

Based on the three modalities Speech, Gesture, and Touch, we developed six interaction techniques for the interaction task Visual Inspection. For each modality two variants were conceptualized to increase diversity. They are illustrated in Figure 2 and briefly outlined below.

##### Artificial Operator (AO)

AO builds upon the metaphor of an assisting human operator. The speech recognizer handles filler and stop words to facilitate the feeling of a natural conversation. Free navigation requiring fine-tuning of numerical values, e.g., the view angle, is inconvenient due to the nature of human speech. Instead, a set of predefined verbal command patterns are available, which are derived from analyzing conventional design reviews and capture a wide variety of vocabulary and syntactic rules, for instance: “show me the rear three quarter view please.”

##### Speech Menu (SM)

SM combines speech input and a graphical menu shown in the top right corner of the VR screen. The menu has a hierarchical structure composed of several sub menus, which is comparable to the structure of context menus of customary PC user interfaces. Each action is mapped to a menu item and executed instantly, when the user has spoken the item text. Similar to AO, the navigation is limited to a predefined set of commands displayed in the menu.

##### Air Touch (AT)

The idea of AT is to operate the large VR screen in the same way as a customary touchscreen without touching it. The user interacts with a virtual touch panel hovering in the air. The interactive area is located in an ergonomically convenient position approximately 35 cm in front of the user's shoulder joint. Touch events are triggered, when hand joints tracked by the 3D camera system exceed the distance threshold with respect to the corresponding shoulder joint. Valid touches are indicated on the VR screen using the left or right hand icon, respectively. Movements of touch points in X or Y direction (one-handed control) cause horizontal or vertical rotation of the virtual camera around the car. For zooming in or out, the user alters the distance between two touch points (two-handed control).

##### First Person (FP)

The FP concept imitates the visual inspection of a physical car exterior. 3D cameras track the users head and link its position permanently with the virtual camera, which always looks at the car. Thus, all movements of user immediately affect the view. That improves the immersion effect significantly. For zooming in or out, the user simply walks toward or away from the VR screen. Moving sideward, kneeling or jumping also adjust the view position in the physically expected manner. Leaning the torso to the left or right causes the virtual camera to horizontally rotate around the car with defined speed that is proportional to the leaning angle. Unfortunately, vertical rotation cannot be implemented in a similar fashion, since it is impossible in the physical world as well.

##### Map View (MV)

The principle of MV is to interact with a schematic 2D map in top view comparable to digital map services like Google Maps^1^. The map is rendered on a mobile tablet PC that acts as a handy remote control for the VR system. All objects the user can interact with are depicted as 2D icons. In order to navigate, the user moves the 2D camera icon or the 2D camera view target icon with one finger. Dragging the camera icon changes the virtual camera location, dragging the camera view target icon changes the view direction of the virtual camera.

##### Direct Touch (DT)

DT allows users to directly navigate on the 2D view of the 3D scene, which is streamed in real time from the main VR screen to a mobile tablet PC with touch screen capabilities. The user navigates by simply dragging one or two fingers anywhere on the touch screen. The horizontal or vertical view angle can be altered by moving the index finger in X or Y direction (1 finger swipe gesture). Zooming is performed by changing the distance between thumb and index finger (2 finger pinch gesture).

#### Implementation

**Figure 3:**
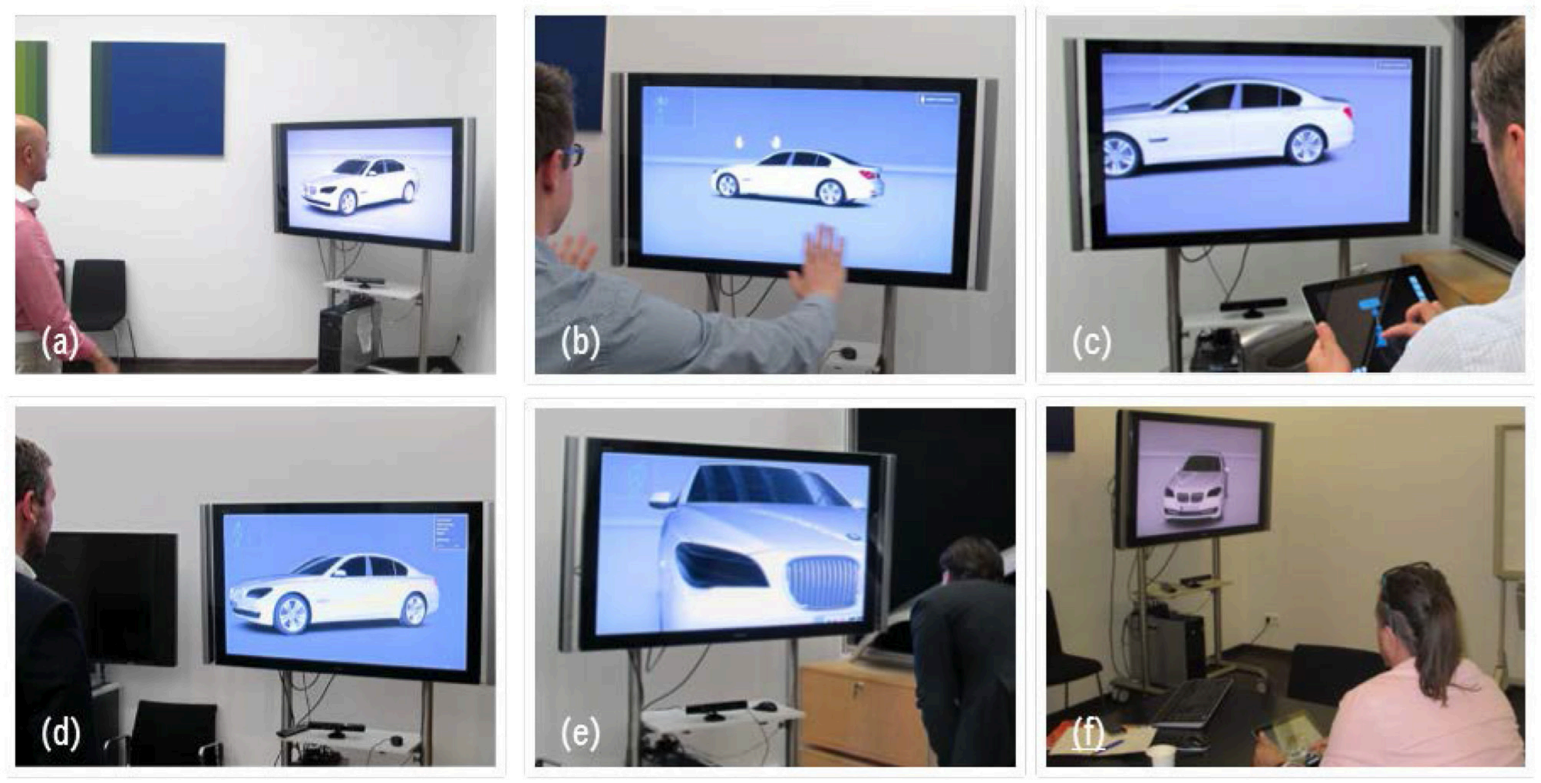
Implemented interaction techniques for Visual Inspection: **(a)** Artificial Operator, **(b)** Air Touch, **(c)** Map View, **(d)** Speech Menu, **(e)** First Person, and **(f)** Direct Touch.

For experiments and proof of technical feasibility, we implemented the interaction concepts into a high fidelity system prototype. Hardware parts are chosen to be compact and are mounted on a mobile metal frame in order to increase flexibility for the user study and limit costs. Figure 3 depicts users interacting with the system prototype.

As simulation software we deploy the 3D game engine 3DVIA Virtools, because we can build on top of a sophisticated BMW shader library and production data import workflow. Moreover, the software also has a flexible graphical scripting language, which enables programming and refining interaction logic efficiently at run time. The scene contains BMW 5 Series production data and runs on a high performance workstation with more than 100 frames per second on average. The rendered image is outputted on a 55” LCD with Full HD resolution (1,920 × 1,080 pixels).

For the speech- and gesture-based interactions techniques as described in the previous section, we use the Microsoft Kinect Sensor, which is a 3D tracking camera system with integrated microphone array. Low level data processing is performed by the Microsoft Kinect SDK for skeletal tracking and Microsoft Speech API for speech recognition. The generated raw data is streamed into the 3D game engine, where the high level interaction logic is implemented with C++ and the Virtools scripting language.

The touch-based interaction techniques are realized using the Apple iPad, which is a high quality tablet PC having multi-touch capabilities. Both concepts “Map View” and “Direct Touch” are programmed as two modes of one holistic mobile application using the native programming environment Xcode. The wireless data communication between the mobile app and the 3D game engine is established with a TCP/IP client-server paradigm. The client (mobile app) sends user inputs to the server (3D game engine), where the high level interaction logic is executed.

### User Study

#### Method

##### Study design and metrics

We choose a within-subjects experimental design and vary the independent variable “interaction technique.” Thus, we have one within factor with six levels (AO, SM, AT, FP, MV, DT). To compensate for order and training effects, the sequence is randomized for each participant. Our randomization scheme prevents consecutive ratings of interaction techniques A and B of the same modality. As dependent variables, we use four subjective compound measures to quantify the perceived quality of use. These are Usability, User Experience, Intuitiveness, and Task Load, since they are widely used in HCI research and can be surveyed by standardized questionnaires.

*Usability*. To characterize the perceived Usability we use the System Usability Scale (SUS) questionnaire, which is one of the most frequently used questionnaires for this purpose (Brooke, 1996). Participants rate ten items, which are combined into a single SUS score ranging from 0 (low usability) to 100 (high usability). All items are worded as statements, five positively and five negatively, and participants choose their degree of assent using a five-point scale. Because of its intensive use in research, there exist generally accepted thresholds for interpretation (Bangor et al., 2009). Scores below 60 indicate bad usability and scores above 80 indicate good usability.

*User Experience*. We employ the AttrakDiff2 questionnaire to investigate the User Experience, which is measured in the dimensions Pragmatic Quality (PQ), Hedonic Quality Identification (HQ-I), Hedonic Quality Stimulation (HQ-S), and Attractiveness (ATT) (Hassenzahl and Monk, 2010). The measurement scale of each dimension ranges from 1 (low quality) to 7 (high quality). We choose the short Mini-AttrakDiff2 with only seven items instead of the standard questionnaire with 28 items to unload participants and reduce processing time. The items represent a semantic differential that employs a seven anchor scale with a pair of bipolar attributes (e.g., “simple” vs. “complicated”, “cheap” vs. “valuable”, “ugly” vs. “beautiful”).

*Intuitiveness*. To quantify how intuitive the interaction in VR is experienced, the participants fill in the INTUI questionnaire (Ullrich and Diefenbach, 2010). Intuitiveness is a compound metric based on four independent dimensions, which are Effortlessness (E), Gut Feeling (GF), Magical Experience (ME), and Verbalization (V). The values vary between 1 (low characteristic) and 7 (high characteristic). In total the questionnaire constitutes of 17 questions whereby participants decide between two contradicting statements on a seven-point scale (e.g., “Using the product was very intuitive” vs. “Using the product wasn't intuitive at all”).

*Task Load*. The NASA-TLX questionnaire provides a measure of the perceived Task Load based on six subscales reflecting the mental, physical, and temporal load as well as the performance, effort, and frustration level (Hart and Staveland, 1988). To each subscale one questions is assigned (e.g., “How mentally demanding was the task?”) and the degree of assent is measured on a numerical scale between 0 and 100 and five-point step width. The end points are defined by verbal anchors (“very low” vs. “very high”). All subscales are equally weighted in order minimize processing time and aggregated into the overall task load index (TLX) ranging from 0 (low task load) to 100 (high task load).

##### Procedure

Each participant grades all six interaction techniques sequentially. At the beginning the examiner briefly introduces the study goal and procedure. Then the participant is asked to answer a demographic questionnaire. After the introductory phase, the participant is confronted with the first interaction technique. The examiner explains its usage and concedes a settle-in period. As soon as the participant feels familiar with the interaction technique, he or she is requested to accomplish the interaction task “find and document design problems.” This task mimics typical usage behavior during design reviews and consists of two steps, which are (1) to navigate to certain exterior views requested randomly by the examiner and (2) to take screenshots of them. Eventually, the participant is asked to fill in the questionnaires measuring the dependent variables. After the test run is completed, the examiner proceeds with the next condition until all interaction techniques are evaluated. One condition lasted about 10–15 min and the entire study took 90 min on average.

##### Participants

The participants were recruited using e-mail and personal acquisition. Twenty-four participants (6 female, 18 male) aged between 20 and 56 years (*M* = 31.1, *SD* = 10.1) took part in the user study. All had normal or corrected to normal visual acuity and no impairment to physical and mental health. One half of the participants were professionals from BMW Group and the other half were students from Fraunhofer IAO. We deliberately opted for this partitioning, as the results specifically should reflect users with only few VR experience and processual knowledge.

#### Results

##### Quantitative results

Table 1 shows a summary of the results. For analyzing each metric, Repeated Measures Analysis Of Variance (RM-ANOVA) is conducted, since histograms and P-P plots indicate that the data is normally distributed. Violations of the sphericity assumption tested by a Mauchly-Test are addressed by adjusting the degrees of freedom and *p*-value with Greenhouse-Geisser or Huynh-Feldt epsilon, respectively. *Post-hoc* comparisons are calculated using Bonferroni corrected pairwise *t*-tests. The significance level is set to α = 5%.

**Table 1:** Means and 95% confidence intervals of all metrics and interaction techniques.

	**Artificial Operator**	**Speech Menu**	**Air Touch**	**First Person**	**Map View**	**Direct Touch**
SUS^***^	55.10 ± 9.05	71.67 ± 5.83	72.92 ± 7.81	78.65 ± 6.62	72.40 ± 8.34	**79.06** **±** **9.06**
PQ^***^	4.22 ± 0.62	5.23 ± 0.38	5.03 ± 0.56	**5.71** **±** **0.34**	5.25 ± 0.49	5.68 ± 0.49
HQ-I^***^	4.81 ± 0.43	4.50 ± 0.43	5.25 ± 0.41	5.25 ± 0.43	5.25 ± 0.37	**5.44** **±** **0.38**
HQ-S^*^	4.88 ± 0.51	4.38 ± 0.36	4.96 ± 0.42	5.06 ± 0.53	**5.15** **±** **0.41**	4.71 ± 0.49
ATT^**^	4.54 ± 0.64	4.92 ± 0.40	5.25 ± 0.45	5.29 ± 0.50	5.44 ± 0.44	**5.77** **±** **0.37**
E^***^	4.01 ± 0.61	5.02 ± 0.48	4.97 ± 0.57	5.48 ± 0.43	4.90 ± 0.62	**5.62** **±** **0.59**
GF^***^	3.73 ± 0.56	2.90 ± 0.50	4.22 ± 0.64	**4.86** **±** **0.60**	3.84 ± 0.50	**4.86** **±** **0.62**
ME	4.73 ± 0.44	4.69 ± 0.46	5.05 ± 0.39	5.22 ± 0.50	4.97 ± 0.39	5.18 ± 0.40
V^**^	4.91 ± 0.61	5.94 ± 0.41	5.68 ± 0.47	**6.01** **±** **0.50**	5.15 ± 0.56	5.82 ± 0.55
TLX^***^	38.65 ± 6.73	32.67 ± 6.57	35.52 ± 8.14	26.32 ± 5.66	27.33 ± 6.37	**18.75** **±** **5.00**

*The best rated concept of each metric is highlighted (bold font)*.

* *p < 0.05*,

** *p < 0.01*, and

*** *p < 0.001*.

*Usability*. System Usability Scale (SUS) reveals statistically significant differences: *F*_(4.662, 107.237)_ = 7.278, *p* < 0.001. “Direct Touch” is perceived to have the highest Usability, which is significantly higher than “Artificial Operator” (p_DT−AO_ = 0.001).

*User experience*. Pragmatic Quality (PQ) between the interaction techniques differ significantly: *F*_(3.678, 84.586)_ = 6.341, *p* < 0.001. “First Person” and “Direct Touch” show best ratings and are significantly more pragmatic than “Artificial Operator” (p_FP−AO_ < 0.001, p_DT−AO_ = 0.001).

Hedonic Quality Identification (HQ-I) has significant differences: *F*_(5, 115)_ = 4.909, *p* < 0.001. The best rating can be reported for “Direct Touch” that significantly outperforms “Speech Menu” (p_DT−SM_ < 0.001).

Hedonic Quality Stimulation (HQ-S) reveals statistical significances between the interaction techniques: *F*_(5, 115)_ = 2.790, *p* = 0.020. “Map View” receives highest grading and is significantly better than “Speech Menu” (p_MV−SM_ = 0.020).

Significant differences can also be found for Attractiveness (ATT): *F*_(3.310, 76.141)_ = 4.880, *p* = 0.003. “Direct Touch” shows the highest score. Pairwise comparisons prove, that it is significantly more attractive than both speech-based concepts (p_DT−AO_ = 0.003, p_DT−SM_ = 0.004).

*Intuitiveness*. Effortlessness (E) shows significant differences between the interaction techniques: *F*_(5, 115)_ = 5.216, *p* < 0.001. “Direct Touch” and “First Person” yield best ratings and require significantly less effort than “Artificial Operator” (p_DT−AO_ = 0.001, p_FP−AO_ = 0.001).

Gut Feeling (GE) unveils significant differences: *F*_(3.241, 74.537)_ = 9.416, *p* < 0.001. “Direct Touch” and “First Person” equally show best results and both are perceived significantly better than “Map View” and “Speech Menu” (p_DT−MV_ = 0.001, p_DT−SM_ = 0.023, p_FP−MV_ = 0.031, p_FP−SM_ = 0.001).

Magical Experience (ME) does not show significances: *F*_(3.152, 72.502)_ = 1.820, *p* = 0.148.

Verbalization (V) is statistically significant: *F*_(5, 115)_ = 4.464, *p* = 0.001. “First Person” receives the highest rating, which is significantly higher than “Artificial Operator” (p_FP−AO_ = 0.024).

*Task Load*. Overall Task Load Index (TLX) shows significant differences: *F*_(5, 115)_ = 6.841, *p* < 0.001. “Direct Touch” causes the lowest task load, which is significantly lower than the task load of both speech-based interaction techniques and “Air Touch” (p_DT−AO_ < 0.001, p_DT−SM_ = 0.004, p_DT−AT_ = 0.014). Furthermore, “First Person” induces a significantly lower task load than “Artificial Operator” (p_FP−AO_ = 0.013).

##### Qualitative results

The statements and comments of the participants match with the quantitative results. The majority emphasized the simplicity and ease of use of “Direct Touch” (16 participants) and “First Person” (16 participants) which reflects their positive ratings overall. While using the speech-based techniques the participants focused more on the deficiencies. Especially, the criticism aimed at the mental effort to learn the possible phrases of “Artificial Operator” (11 participants) and the unfamiliar menu control of “Speech Menu” (19 participants). In addition, the ratings of both speech concepts suffered from the recognition error rate that was perceived as too high (AO: 11 participants, SM: 5 participants). As captured by the questionnaires, the participants stated various pros and cons of “Air Touch” and “Map View” without clear tendency compared to the other techniques. Altogether, the participants uniformly confirmed the necessity to directly interact with the virtual design model and welcomed the diversity and quality of the implemented interaction concepts.

#### Discussion

The touch-based “Direct Touch” and gesture-based “First Person” received best ratings in terms of Usability, User Experience, Intuitiveness, and Task Load. Regarding these aspects both interaction techniques are rated significantly above average with respect to the corresponding scale of measurement. As compared to “Air Touch” and “Map View” the ratings are mostly better, but differences are rarely significant. However, as compared to the speech-based concepts “Artificial Operator” and “Speech Menu” the ratings are significantly better for many dimensions. “Direct Touch” and “First Person” show overall highest usage quality among the presented interaction techniques and are appropriate candidates for further refinement loops.

Our study has the following limitations. First, we exclusively focus on vehicle exteriors. Our approach does not incorporate requirements of the interior assessment (e.g., sense of space, seating position, haptic feedback, operating car UI), which are completely distinct and have to be examined separately. Second, the study was conducted using a 55″ display in monoscopic mode. Although this is a common system setup for ordinary design reviews, it only represents a technological subset. The study does not capture the effect of other common display types (e.g., powerwall, stereoscopic view). Third, the usage scenario even though closely related to real industrial settings still is artificial. While this increases internal validity because the study conditions can be fully controlled, it reduces external validity.

## Iteration 2: Interaction Techniques for Model Comparison

In the second iteration, we focused on interaction techniques to support the required functionality of the second main interaction task Model Comparison. Proceeding from previous findings, we refined the concepts, implemented a high fidelity prototype and reevaluated the perceived quality of use.

## Concept and Implementation

### Concept

**Figure 4:**
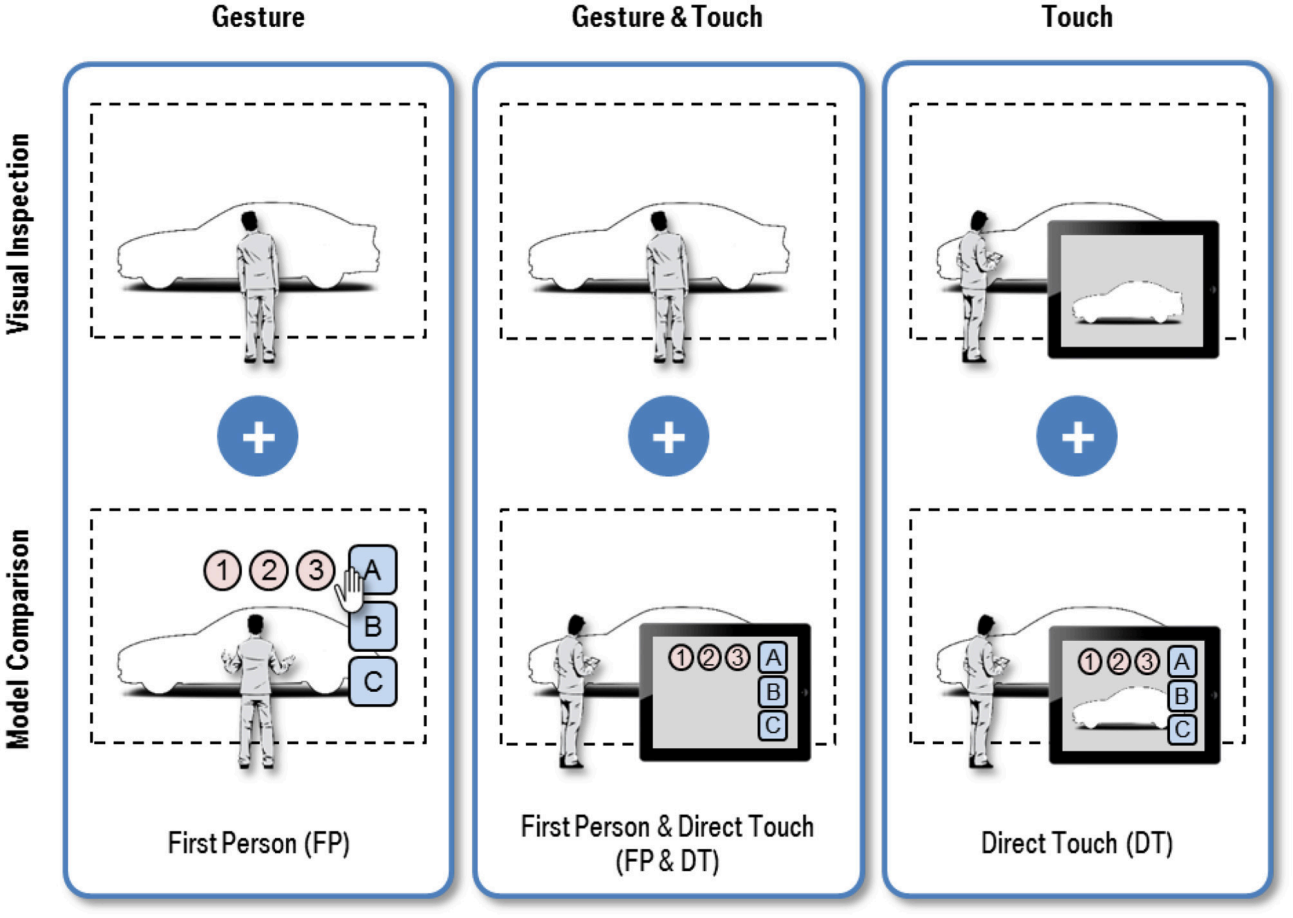
Interaction techniques for Visual Inspection and Model Comparison of 3D vehicle exteriors.

The two best rated interaction techniques of the first iteration, the gesture-based “First Person” and touch-based “Direct Touch,” serve as starting point for refinement. Using the same input modality we expanded both concepts by functionality to compare model variants. Additionally, we introduce the hybrid concept “First Person and Direct Touch,” which represents a multimodal mixture of both combining Gesture and Touch input modalities. Figure 4 gives a schematic overview.

#### First Person (FP)

FP is intended to mimic the visual inspection of cars in the real world. To enable users to compare vehicle variants, a hand gesture controlled 2D GUI is introduced. The mechanics of GUI interaction builds closely on the ideas of the previous concept AT. By default, the GUI is hidden to not obscure the exterior model and fades in when the hand of the user touches the virtual touchscreen. Depending on which hand is used, the GUI appears on the left or right border of the VR screen. While the GUI is visible, navigation is suspended to prevent operating errors. Similar to operating an ordinary computer by mouse, the hand is connected to a virtual 2D pointer indicated by a hand icon. The user activates graphical widgets by simply grabbing it, i.e., closing and opening the fingers which is visually reflected by animating the hand icon. The interaction technique is based on hand and body movements tracked by 3D cameras.

#### Direct Touch (DT)

DT enables users to visually inspect vehicle exteriors by touching a real-time rendering of the 3D VR scene on a mobile touch device. The functionality to switch between variants is implemented using a 2D GUI that overlays the rendered image when activated. The look and feel of operating the GUI mirrors the GUI implemented in the refinement of FP presented in the previous section and behaves like common mobile applications. The GUI is invisible under normal operations and fades in or out when the user presses a button widget in the top border of the display. If the GUI is rendered, the navigation mode is locked which is indicated by darkening the 3D scene on the mobile device to prevent unintended misuse. The concept builds on the Touch input modality and a portable touch display with pad dimensions as remote control.

#### First Person and Direct Touch (FP&DT)

In addition, we developed the new hybrid interaction technique FP&DT, which is a multimodal combination of FP and DT. The functionality of Visual Inspection corresponds exactly to the implementation of FP. For Model Comparison the concepts provides a 2D GUI on mobile touch device. The GUI containing all variants is shown permanently on the device. In contrast to the refinement of DT, no real-time view of the 3D scene is streamed to the device, to prevent the user from distraction. The multimodal concept incorporates Gesture and Touch input modality simultaneously using 3D cameras for tracking and a mobile touch device as remote control.

### Implementation

**Figure 5:**
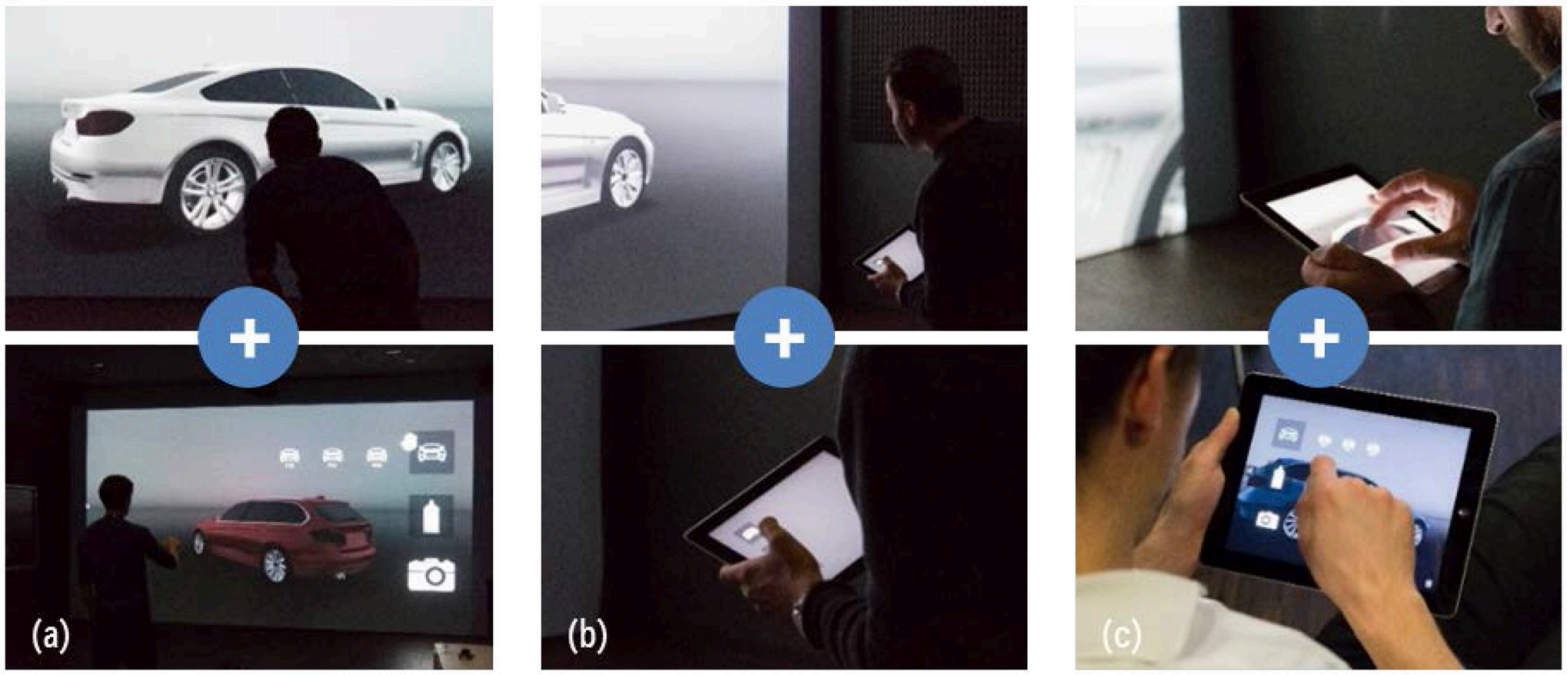
Implemented interaction techniques for Visual Inspection (top row) and Model Comparison (bottom row): **(a)** First Person, **(b)** First Person and Direct Touch, and **(c)** Direct Touch.

To account for potential effects of immersion as discussed in the previous study, we integrated the three concepts into a powerwall system located at the BMW design department in Munich. This type of setup is widely used in the automotive industry and therefore offers realistic usage conditions (Küderli, 2007; Zimmermann, 2008). Participants using the interaction techniques are shown in Figure 5.

We use the 3D game engine Unity 3D as simulation framework. It is well-suited for rapid prototyping in VR, as it ships with the versatile programming language C#.NET and integrates most third-party modules easily. In order to showcase the interaction capabilities, three exterior geometry variants (sedan, coupé, station wagon) and three color variants for each geometry (silver, red, blue) are prepared in the scene and optimized to perform at least at 100 frames per second on an ordinary workstation. The powerwall setup consists of a 4 k projector (4,096 × 2,160 pixels) in front projection mode and a large canvas (6 × 4 m) allowing for real scale visualizations.

The gesture-based “First Person” processes hand and body movements. To enable natural interaction without attaching physical sensors to the user, we exploit the Microsoft Kinect Sensor as low-cost tracking device. 3D joint positions for navigation and open or close state of the hand for GUI interaction are reliably recognized by the Microsoft Kinect SDK in real-time. As the SDK integrates seamlessly in Unity 3D, the high level interaction logic is programmed entirely with C#.NET scripts.

For the touch-based “Direct Touch,” we use the Apple iPad as a portable touch remote. Wireless data connection between the workstation and mobile device is handled using a TCP/IP-based client-server model. The mobile app (client) is programmed completely within the Unity 3D framework, since it enables software development for many platforms. The simulation (server) receives user input events from the mobile app, executes the interaction logic, and feeds back the real-time video stream to the mobile app.

The implementation of the hybrid “First Person and Direct Touch” builds on top of this infrastructure including appropriate extensions in related software parts.

## User Study

### Method

#### Study design and metrics

We replicate the experimental design of the first user study. But as a result of the previous discussion, we incorporate improvements. Again, varying the independent variable “interaction technique” results in a within-subjects design with one within factor composed of three levels (FP, DT, FP&DT). To attenuate order and training effects, we permute the sequence of interaction techniques for each participant. Having a sample size of twenty-four and six possible permutations, there are six groups with four subjects testing the same sequence. We also reuse the four compound qualities Usability, User Experience, Intuitiveness, and Task Load as dependent variables (Hart and Staveland, 1988; Brooke, 1996; Hassenzahl and Monk, 2010; Ullrich and Diefenbach, 2010).

#### Procedure

Similar to previous procedure, each participant tests all three interaction techniques one by one. After the examiner explained the study goals and procedure, the participant fills in a demographic questionnaire. Depending on the sequence group, the participant is assigned the first concept. Following a brief demonstration and settle-in period, the examiner asks the participant to find and take screenshots of car paint scratches. To each of the nine vehicle variants (three geometry variants times three color variants) one scratch is attached at one random location using a “random scratch generator.” This setup allows the participant to autonomously explore all concepts, which facilitates a profound evaluation and increases objectivity, as the examiner is less involved. This task simultaneously combines the two main tasks Visual Inspection (“find scratch on current vehicle”) and Model Comparison (“switch to a specific variant”). Finally, the participant answers the questionnaires measuring the dependent variables and the procedure continues with the next concept. On average, the participants spent 10–15 min testing each interaction technique and the total procedure took about 45 min per subject.

#### Participants

The sample consisted of 24 subjects (9 female, 15 male), who were asked personally and per e-mail to join the study. No subject took part in the previous study. The age ranged between 21 and 59 years (*M* = 35.4, *SD* = 9.7). All participants had normal or corrected to normal visual acuity and normal physical and mental health. In the second iteration, we choose all participants to be design professionals from BMW Group, as we shift the study emphasis on more realistic test conditions.

### Results

#### Quantitative results

The findings are summarized in Table 2. Again, since histograms and P-P plots suggest that the data is normally distributed, we reused the previous analysis approach and significance level α = 5%.

**Table 2:** Means and 95 % confidence intervals of all metrics and interaction techniques.

	**First Person**	**First Person and Direct Touch**	**Direct Touch**
SUS^*^	71.77 ± 8.53	78.96 ± 7.73	**86.46** **±** **7.58**
PQ^*^	5.18 ± 0.58	5.68 ± 0.42	**6.08** **±** **0.44**
HQ-I	4.90 ± 0.47	4.94 ± 0.42	5.15 ± 0.47
HQ-S^**^	**5.38** **±** **0.40**	5.27 ± 0.41	4.46 ± 0.54
ATT	5.19 ± 0.55	5.44 ± 0.47	5.31 ± 0.47
E^*^	5.06 ± 0.62	5.80 ± 0.50	**5.97** **±** **0.50**
GF	3.71 ± 0.60	4.07 ± 0.59	4.53 ± 0.60
ME^***^	**5.22** **±** **0.44**	4.89 ± 0.48	4.00 ± 0.53
V^*^	5.96 ± 0.49	6.40 ± 0.32	**6.45** **±** **0.39**
TLX^***^	29.13 ± 7.67	22.12 ± 6.20	**14.55** **±** **4.33**

*The best rated concept of each metric is highlighted (bold font)*.

* *p < 0.05*,

** p < 0.01, and

*** *p < 0.001*.

*Usability*. Although the concepts show a significant effect on System Usability Scale (SUS), pairwise comparisons do not elucidate individual differences: *F*_(1.711, 39.350)_ = 4.221, *p* = 0.027. This indicates an existing but small effect size in terms of Usability.

*User Experience*. The interaction techniques have high impact on Hedonic Quality Simulation (HQ-S): *F*_(1.489, 34.246)_ = 10.084, *p* = 0.001. Pairwise comparisons prove that “First Person” (*p* = 0.004) and “First Person and Direct Touch” (*p* = 0.014) are perceived significantly more stimulating than “Direct Touch.” However, the difference between “First Person” and “First Person and Direct Touch” is not significant. Therefore, the influence is mostly due to navigation based on the body movements.

Similar to Usability, the effect on Pragmatic Quality (PQ) is significant, but pairwise comparisons do not confirm any individual differences: *F*_(1.388, 31.917)_ = 4.799, *p* = 0.025.

In contrast, Hedonic Quality Identification (HQ-I) and Attractiveness (ATT) do not imply significant results: HQ-I: *F*_(2, 46)_ = 0.783, p = 0.463; ATT: *F*_(2, 46)_ = 0.353, *p* = 0.705.

*Intuitiveness*. The results expose a strong effect of the concepts on Magical Experience (ME): *F*_(1.493, 34.330)_ = 11.172, *p* < 0.001. “First Person” (*p* = 0.003) and “First Person and Direct Touch” (*p* = 0.013) are perceived significantly more exiting compared to “Direct Touch.” As the difference between “First Person” and “First Person and Direct Touch” is not statistically relevant, the effect is likely caused by the body-controlled navigation method.

Effortlessness (E) and Verbalization (V) both reveal significant results, but pairwise comparisons do not expose further insight: E: *F*_(2, 46)_ = 4.110, *p* = 0.023; V: *F*_(1.703, 39.164)_ = 3.862, *p* = 0.036.

Finally, the interaction techniques have no influence on the attribute Gut Feeling (GF): *F*_(2, 46)_ = 2.993, *p* = 0.060.

*Task load*. The Overall Task Load Index (TLX) shows significant impact of the interaction techniques: *F*_(2, 46)_ = 9.497, *p* < 0.001. The pairwise comparisons reveal a lower perceived task load using “Direct Touch” compared to “First Person” (*p* = 0.003). Whereas, differences of “First Person and Direct Touch” compared to “First Person” (*p* = 0.070) and “Direct Touch” (*p* = 0.083) are not significant.

##### Qualitative results

The results of the questionnaires are validated by user statements and comments. The participants pointed out the simplicity and ease of use of the touch-based “Direct Touch” as in the previous study (23 participants). On the flip side, nine participants expressed the lack of functionality (e.g., pan in view plane, change focus point) and eight participants remarked that the interaction was “not compelling.” Regarding the gesture-based “First Person,” the participants appreciated the simplicity (16 participants), the natural impression using body and hand movements (15 participants), and the positive emotional experience (8 participants). In contrast, the difficult menu control with hand gestures (18 participants), mental effort (12 participants), and physical effort (11 participants) were stated as weak points. Lastly, the hybrid “First Person and Direct Touch” shares the same advantages and disadvantages of both concepts with almost equal weightings.

## Discussion

All three interaction techniques were rated decidedly above average compared to scales of measurement. Regarding rational attributes (SUS, PQ, E, V, TLX) the refined touch-based “Direct Touch” was perceived best. In contrast, the participants experienced emotional attributes (HQ-S, ME) of the refined gesture-based “First Person” more pronounced. The multimodal “First Person and Direct Touch” inherits the qualities of the better rated concept, but slightly less intense. In summary, all concepts seem ideally suited for VR-based Automotive Design Reviews. However, the optimal choice depends on specific use case characteristics and individual user preferences.

To overcome some of the discussed limitations of the previous user study, we made three modifications. First, we integrated all concepts into one immersive powerwall system, which represents the de facto standard in automotive design and therefore enhances external validity. However, because the mobile app only supports monoscopic mode, the study does not address the impact of stereoscopic view. Second, the sample consisted entirely of BMW design professionals. In comparison with the previous mixed sample, the results primarily convey perceptions of potential users, which also contributes to external validity. Third, the modified interaction task empowers autonomous operations. By reducing the involvement of the examiner and concentrating the attention on the interaction techniques, we encourage more profound and objective ratings.

## Conclusion and Outlook

**Figure 6:**
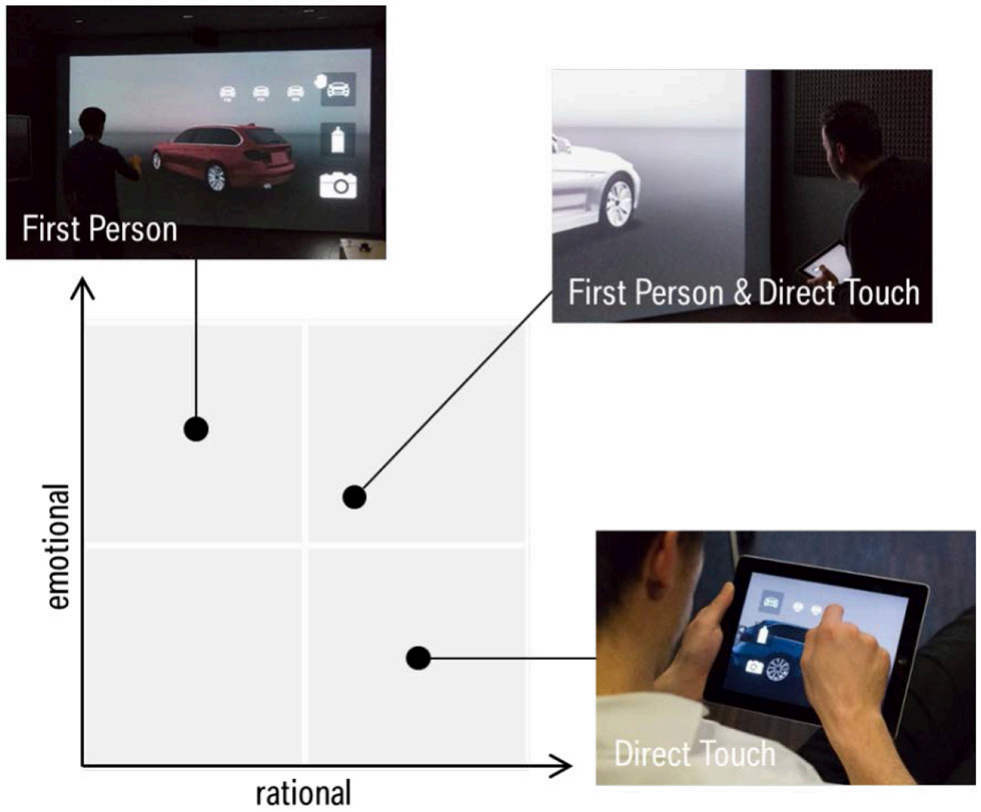
Characteristics of interaction techniques regarding rational and emotional qualities.

In this paper, we investigated interaction techniques for the design assessment of automotive exteriors. Our user centered approach comprised of two iterations studying two interaction tasks: Visual Inspection and Model Comparison. In each iteration, we developed solution concepts and implemented high fidelity prototypes. Conducting user studies, we examined the influence of the interaction techniques on the perceived Usability, User Experience, Intuitiveness, and Task Load. Altogether, the results confirm that the gesture-based “First Person,” touch-based “Direct Touch,” and hybrid “First Person and Direct Touch” provide high quality of use, but reveal significantly distinct user perceptions regarding rational and emotional characteristics. These characteristics are schematically depicted in Figure 6.

Despite the discussed limitations of our approach, the outcomes show that the VR-based Automotive Design Review would benefit from these interaction techniques. Beyond that, the transfer to other closely related use cases (e.g., Car Clinic, Auto Show, Point of Sale) seems promising and demands further investigation:

- The **Car Clinic** is a standard market research method to systematically measure the subjective opinion of customers on new vehicles. For VR-based setups, we suggest the hybrid “First Person and Direct Touch” to reduce the training period for the participants, since it allows for natural body movements to visually inspect the car and intuitive touch controls to switch car variants. Furthermore, the touch remote facilitates the completion of research questionnaires.
- **Auto Shows** are used by car manufactures to advertise new vehicles and collect useful feedback from public media. Innovative exhibition stands using newest VR technology help to provide the visitors with a positive product experience. Based on our study results, we think that adapting the gesture-based “First Person” contributes to the emotional characteristic of those events.
- The **Point of Sale** increasingly shifts from the outskirt to the privileged city center due to changing customer behavior. In this scenario, VR enables car dealers to promote the entire product portfolio despite limited floor space. Our study results indicate that on the one hand the gesture-based “First Person” increases the customer experience and on the other hand the touch-based “Direct Touch” supports the sales pitch.

In future we plan to industrialize the interaction techniques and study the performance under real conditions. Using the example of an ordinary design review, we want to optimize for daily use in industrial settings. Moreover, we plan to merge the interaction techniques into a multimodal user interface (Oviatt, 2012; Turk, 2014). Offering such a user interface to designers, engineers and management executives would substantially increase the acceptance of VR in automotive design and thus, contribute to the digitization of the automotive industry.

## Ethics Statement

The paper describes a usability study with humans as participants within a BMW Group research project. All participants were kindly asked to join, but nobody was forced to join by any means. Every participant was fully informed about the goals and procedure in the beginning and able to quit the study at any time. The data was surveyed completely anonymously using questionnaires and it is impossible to assign results to particular subjects.

## Author Contributions

All authors listed have made a substantial, direct and intellectual contribution to the work, and approved it for publication.

### Conflict of Interest Statement

The authors declare that the research was conducted in the absence of any commercial or financial relationships that could be construed as a potential conflict of interest.

## Footnotes

^1^Available online at: http://www.google.de/maps (Accessed Sept, 29, 2017).
